# Paraspinal Muscle Stiffness during Hamstring Exercise Using Shear-Wave Elastography

**DOI:** 10.3390/sports12080199

**Published:** 2024-07-23

**Authors:** Eleftherios Kellis, Afxentios Kekelekis, Eleni E. Drakonaki

**Affiliations:** 1 Laboratory of Neuromechanics, Department of Physical Education and Sport Sciences at Serres, Aristotle University of Thessaloniki, Agios Ioannis, 62110 Serres, Greece; afxentio@phed-sr.auth.gr; 2 Department of Anatomy, Medical School, University of Crete, 71110 Heraklion Crete, Greece; drakonakielena@gmail.com

**Keywords:** spine, elastogram, stretching, isometric, semimembranosus, remote exercise effect

## Abstract

Soccer teams integrate specific exercises into their typical workout programs for injury prevention. This study examined the effects of hamstring exercise on paraspinal and hamstring stiffness. These findings can inform training and rehabilitation programs to improve muscle health and prevent injuries. Fifteen young, healthy males performed passive and active (submaximal) knee flexion efforts from 0°, 45°, to 90° angle of knee flexion from the prone position. Using shear-wave elastography (SWE) and surface electromyography, we measured the elastic modulus and root mean square (RMS) signal of the erector spinae (ES), multifidus (MF), semitendinosus (ST), and semimembranosus (SM) during different knee flexion angles. Passive SWE modulus at 0° was 12.44 ± 4.45 kPa (ES), 13.35 ± 6.12 kPa (MF), 22.01 ± 4.68 kPa (ST), and 21.57 ± 5.22 kPa (SM) and it was greater (*p* < 0.05) compared to 45° and 90°. The corresponding values during knee flexion contractions at 0° increased to 18.99 ± 6.11 kPa (ES), 20.65 ± 11.31 kPa (MF), 71.21 ± 13.88 kPa (ST), and 70.20 ± 14.29 kPa (SM) and did not differ between angles (*p* > 0.05). Compared to rest, the relative increase in the SWE modulus during active contraction had a median value (interquartile range) ranging from 68.11 (86.29) to 101.69 (54.33)% for the paraspinal muscles and it was moderately to strongly correlated (r > 0.672) with the corresponding increase of the hamstring muscles [ranging from 225.94 (114.72) to 463.16 (185.16)%]. The RMS signal was greater during active compared to passive conditions, and it was lower at 90° compared to 45° (for SM/ST) and 0° (for all muscles). The association between paraspinal and hamstring passive muscle stiffness indicates a potential transmission of forces through myofascial connections between the lumbar spine and the lower limbs. In this laboratory setting, hamstring exercises affected the stiffness of the paraspinal muscles.

## 1. Introduction

Studies have reported that exercises or therapeutic interventions in one area of the body can affect muscle function in distant areas due to connections between fasciae and muscles forming force transmission paths [1,2,3,4,5]. These remote effects of exercise have been attributed to connections that exist between fasciae and muscles and form force transmission paths [6].

One of the force transmission paths, which is often referred to as the posterior myofascial chain, is formed by the serial connection between the paraspinal, posterior thigh, tibial and plantar muscles, and fasciae [1,6,7,8]. The functional significance of this myofascial path has been supported by several studies [5,9,10,11]. The posterior myofascial chain, connecting muscles and fasciae from the lumbar spine to the feet, is crucial for load transfer during activities such as running and jumping [5,9,10,11]. In a cadaveric experiment, it has been shown that traction to the biceps femoris (long head) resulted in the displacement of the thoracolumbar fascia, which surrounds the paraspinal muscles [9]. To the best of our knowledge, evidence for the remainder of the hamstrings, such as the semimembranosus (SM) and semitendinosus (ST), is missing. There is, however, anatomical and clinical evidence that supports a potential association between the function of all hamstrings (not just the biceps femoris) and paraspinal muscles. Particularly, the semitendinosus (ST) and long head of the biceps femoris share a common proximal tendon, which is linked with the sacrotuberous ligament and then with the thoracolumbar fascia [12]. In addition, all hamstrings are covered by similar deep fascia [13], which allows force to be transmitted between all synergist hamstring muscles [14]. Further, myofascial release maneuvers, which are applied to the lumbar and the plantar fascia area, influence hamstring flexibility [5] and ankle dorsiflexion resulting in the displacement of the SM [10], while spinal manipulation had an immediate effect on hamstring force production [15]. Further, there is evidence that lumbar muscle length is associated with the strength of the hip extensors in patients with low back pain [16]. Mathematical simulation of running movement has shown that changes in the function of the erector spinae (ES) influence hamstring muscle function [11]. These findings, however, have not been experimentally verified in human muscles.

Transmission of forces between serially connected myofascial units is achieved by changes in the stiffness of the involved musculature [1,17]. Shear-wave elastography (SWE) allows reliable quantification of tissue stiffness from ultrasound images [18], and it has been applied to quantify hamstring and paraspinal muscle stiffness. During static knee extension stretches, there is an increase in hamstring stiffness, which tends to be greater in SM than in ST [19,20,21,22]. During active knee flexion contractions, however, a greater ST SWE modulus compared to SM has been found [23,24]. To the best of our knowledge, no alterations in paraspinal muscle stiffness during passive and active knee flexions have been previously reported. Research has shown that ES and multifidus (MF) elastic modulus increases from the prone to the seated position [25] and continues to increase in the upright and more forward positions of the trunk [26,27], but stiffness differences between ES and MF vary between studies [25,27,28,29,30]. Even though there are suggestions that hamstring muscle function, such as flexibility, correlates with lumbar function [7,16], a recent systematic review has reported inconsistent evidence on the association between low back pain and hamstring flexibility field test scores [31]. Therefore, examination of changes in stiffness of both hamstrings and paraspinal lumbar muscles is worthwhile.

The purpose of this study was to examine the effects of hamstring exercise on paraspinal and hamstring passive and active muscle stiffness. The hypotheses tested were that (a) passive alteration of knee flexion angle influences paraspinal and hamstring muscle stiffness and activation, (b) hamstring contraction affects paraspinal and hamstring muscle stiffness and activation, and (c) the SWE modulus will differ between either the paraspinal (ES, MF) muscles or between the hamstring (ST, SM) muscles.

## 2. Materials and Methods

### 2.1. Participants

Fifteen healthy males (age: 23.1 ± 3.21 years; mass: 77.3 ± 4.11 kg; height: 1.84 ± 0.48 m) participated in this study. Participants included young (less than 30 years), physically active males who trained three times a week for the past year. Participants who were more than 30 years of age, had recent musculoskeletal injuries, surgeries, neurological or inflammatory diseases, or low back pain or musculoskeletal pain, especially in the posterior thigh region, were excluded from the study. The protocol was approved by the Institutional Ethics Committee (ERC/018/2022). Informed written consent was obtained prior to testing.

The minimum sample size that was required to achieve enough statistical power [32] was estimated using G-power (v. 3.1.9.4, Heinrich-Heine-Universität Düsseldorf, Düsseldorf, Germany; http://www.gpower.hhu.de/ (assessed on 21 July 2024)) for a repeated measures analysis of variance. To achieve this, an estimate of the effect size is necessary. This estimate was based on the partial eta squared (η^2^) value. The η^2^ is characterized as small (η^2^ = 0.01−0.05), medium (η^2^ = 0.06−0.13), or large (η^2^ > 0.14) [32]. Hence, for the purpose of this experiment, we used a medium effect size (η^2^ = 0.09) to calculate an effect size of 0.31. Subsequently, the sample size was calculated using the following inputs: effect size = 0.31, power = 0.80, and type 1 error = 5%. The model yielded a sample size of 15 participants.

### 2.2. Procedures

The participants took the prone position on a physiotherapy bed. Their hips and pelvis were in a neutral position, their trunk in contact with the bed, and their hands on their sides [33]. Any pelvic motion was reduced by attaching a wide elastic strap around the pelvis, while additional straps were used to secure the non-tested leg and the trunk (during maximum knee flexion tests).

First, the participants warmed up with static hamstring stretches followed by 3–4 submaximal hamstring contractions at 90°, 45°, and 0° (=full extension) angle of knee flexion. Joint angles were verified using a standard analog goniometer. Knee flexor maximum voluntary contractions (MVCs) were performed against a hand-held dynamometer (K-Force muscle controller, sampling rate 75 Hz, Kinvent, Montpellier, France). The dynamometer consists of a force sensor that is placed just above the malleolus of the lower leg, and it has high validity and reliability [34]. The distance between the dynamometer placement and the lateral epicondyle was measured, and it was used to calculate the torque exerted around the knee. In each joint position, the participant performed 3 MVC efforts of 5 s each. The maximum torque was taken as the MVC value.

To obtain an EMG reference signal from the paraspinal muscles, the participants also performed MVC trunk extension efforts. From the prone position, participants were instructed to lift their trunk maximally for 5 s against a strap that was placed around their mid-thoracic area (approximately between the 7th and 10th thoracic spinal process) while EMG signals were recorded [35]. The participants performed three repetitions, and the maximum EMG value was used as a reference measure.

The main protocol consisted of SWE and EMG measurements in two conditions: passive and active. In the passive condition, measurements were obtained while the participant was relaxed whilst the knee was held for 5 s at 0, 45, and 90° angular positions in a randomized order. In the active condition, the participant performed submaximal contractions at each angle. The target force level value was preset at 70% MVC, and the participant had approximately 2 s to gradually reach that level and then hold for approximately 5 s. This level of submaximal effort was selected for two reasons: first, to avoid saturation of the SWE modulus readings [23], and second, because this effort level is sufficient to significantly activate target muscles without causing excessive fatigue [36].

All recordings were performed separately for each of the three separate locations (location 1 = ES and MF, location 2 = SM, and location 3 = ST) in randomized order with a ten-minute rest period between them. In each condition, three trials were used and stored for further analysis. In case of errors in the registered SWE readings, such as saturation of elastography signal, the trial was canceled and repeated.

### 2.3. SWE Measurements

To visualize the muscles and to obtain elastography measurements, a LOGIQ E9 ultrasound (R5 version, General Electric, Chicago, IL, USA) system was used. B-mode images were recorded using an ML6-15 (4–15 MHz) linear array probe, and a 9 L (2–8 MHz) 2D linear probe was used for elastography measurements. The Young’s elastic modulus (E) in kilopascals (kPa) was calculated as a function of tissue density (*p*, which is assumed to be 1 g/cm^3^) and velocity of shear waves (V) [37] using the equation E = ρ V^2^.

**Figure 1:**
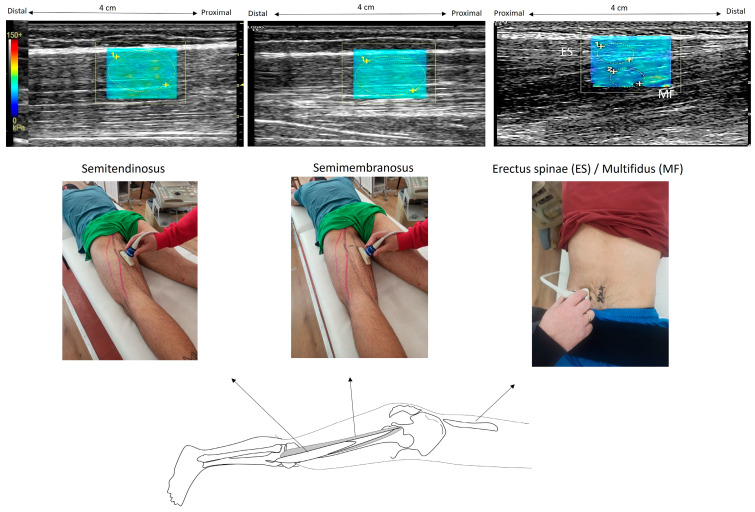
Illustration of elastography measurements. In the prone position, the ultrasound probe was placed on the semitendinosus at 60% of the distance from the ischial tuberosity to the medial condyle; then, it was shifted more medially to capture the semimembranosus. The erector spinae and the deeper region of multifidus were visualized with a probe 4 cm from the L3 spinous process at the L3–L4 level. Example elastography images are also illustrated. Within each elastogram (which appears as a color-coded box), regions of interest were drawn as circles (illustrated with vertical arrows). The software provided the SWE modulus, which was visualized using a color-coded scale. (The color scale was extracted from the software and enlarged so that the measurement scale was easily visible).

The examiner used a B-mode ultrasound to locate the precise locations on the left side of the body and marked them on the skin (Figure 1). To identify the paraspinal muscles, the US probe was rotated transversely to visualize the fourth lumbar (L4) vertebra spinal process, and then the probe was moved laterally to the left, 4 cm away from the L3 spinous process at the L3–L4 level. The ST muscle was visualized by placing the probe at 60% of the distance between the ischial tuberosity and the medial condyle. The probe was then shifted more medially to visualize the SM at the same level. After identifying the level of interest in the axial plane, the probe was shifted longitudinally and parallel to the muscle fibers in order to acquire muscle stiffness measurements. Ultrasound gel was used in all scanned areas to allow clearer US images.

The SWE modulus of the tissues was presented as a rectangular (approximately 4 cm × 3 cm) color-coded box superimposed on each B-mode image, including the subcutaneous fat, the fascia, and the superficial 1 cm of muscle. Recorded modulus values were visualized in real-time in a spectrum that started from blue (very low values), green (low), yellow (intermediate), and finally, red (high). The images were further checked for errors due to signal saturation, and if present, another trial was selected for analysis. Manually selected circular regions of interest (ROIs) were placed on the muscle to obtain the SWE modulus. The same investigator determined the ROIs such that they cover most of the respective muscle area available in the elastogram. Each ROI was approximately 0.5 cm in diameter, and at least 5 ROIs were used within each color-coded box to obtain an average of the SWE readings. The small ROIs were chosen to exclude the fascial planes from each ROI.

For the ES muscle, superficial ROIs were drawn between the aponeurosis of the ES and the epimysial fascia of the MF. For the MF, the ROIs were placed in the deeper region, between the epimysial fascia of the MF and the cortical bone of the mamillary process [25]. For the ST, ROIs were selected from the muscle area, which is located distally to the tendinous inscription. For each trial, three ROIs were obtained in each muscle, and the mean modulus value was calculated. Then, an average SWE modulus of three trials was estimated. All examinations were performed by the same radiologist who has more than 18 years of experience in US and US elastography. To compare the relative increase in the SWE modulus between muscles during contraction, we also calculated the percentage difference in the SWE modulus between rest and contraction (relative SWE modulus) at each joint angle.

### 2.4. EMG Recording and Analysis

In 10 participants (out of the 15 that were evaluated using elastography), EMG activation was obtained simultaneously with the paraspinal muscle SWE recordings. Surface bipolar bar electrodes (TSD 150B, Biopac System Inc., Goleta, CA, USA) with an inter-electrode distance of 1 cm were used to record the EMG signal. A signal receiver and amplifier (TEL100D, Biopac Systems, Inc., Goleta, CA, USA) was interfaced to the electrodes and sampled data using a 12-bit analog-to-digital converter at a 1000 Hz, an input impedance of 10 MΩ, and a common rejection ratio of 130 dB. The signal was multiplied 1000 times and filtered using a band-pass filter (between 15 Hz and 450 Hz) and full-wave rectified. Data were analyzed with the Acknowledge (version 3.9.1, Biopac Systems, Inc., Goleta, CA, USA) software. The root mean square (RMS) was estimated with a 50 ms interval, and it was visible in real time during each test.

For ES measurements, the electrodes were placed on the longissimus lumborum. First, the spinal process of the 1st lumbar ligament (L1) was identified, and the electrodes were placed approximately two-finger widths laterally from the L1 [27]. For MF, the electrodes were placed at the level of the L5 spinous process, 2 cm from the midline. Finally, for the ST/SM, electrodes were placed at 50% of the distance between the medial epicondyle and the ischial tuberosity [27]. Once the positions of the electrodes were marked on the skin, the area was shaved and cleaned with alcohol wipes. A common ground electrode was placed on a bony landmark on the left wrist.

Following EMG data collection, the maximum RMS value, which was recorded during the isometric MVC of each muscle, was stored. Then, the RMS during each testing condition was normalized as a percentage of the MVC value.

### 2.5. Statistical Analysis

Shapiro–Wilk tests showed that MVC torque and RMS data were normally distributed. Hence, differences in the SWE modulus between joint angles (3 angles), conditions (passive, active contraction), and muscles (ES, MF, SM, ST) were examined using a three-way analysis of variance (ANOVA). A two-way ANOVA was used to examine differences in normalized EMG between joint angles (3 angles), conditions (passive, active contraction), and muscles (ES, MF, ST/SM). Differences in MVC torque between angular positions were examined using a one-way ANOVA. Effect sizes were also calculated using the partial eta squared (η^2^) values [32]. If significant, post-hoc Tukey tests were applied to examine significant differences between pairs of means.

The relative SWE modulus data were not normally distributed, and Friedman tests were applied to compare the relative SWE modulus between muscles in each joint angle. The median and interquartile range, which is the difference between the 25th and 75th percentile, of the relative SWE modulus were calculated. In addition, the association of the relative SWE modulus between the four muscles was examined using Spearman’s correlation tests. The correlation was characterized as low if the coefficient (r) was less than 0.3, moderate when 0.3 ≤ r ≤ 0.7, and high when r > 0.7 (based on modification of previous suggestions [38]). Statistical significance was set at *p* < 0.05.

## 3. Results

The MVC torque was 124.65 ± 33.5 Nm at 0°, 121.68 ± 17.01 Nm at 45°, and 107.21 ± 17.84 Nm at 90°. The ANOVA showed a statistically significant effect of angle (F_2,28_ = 4.86, *p* = 0.015, η^2^ = 0.971) on MVC torque. Post-hoc Tukey tests indicated that the MVC torque at 0° and 45° were greater compared to the MVC torque at a 90° angle (*p* < 0.05).

The SWE modulus values for each condition are displayed in Table 1. There was not a statistically significant three-way interaction effect on the SWE modulus (*p* > 0.05). The condition by the muscle (F_3,42_ = 169.47, *p* = 0.0001, η^2^ = 0.924) interaction effect was statistically significant. Post-hoc Tukey tests indicated that both active and passive hamstring muscles’ SWE modulus were greater than paraspinal muscles’ modulus (*p* < 0.05). Further, each muscle modulus was greater during active compared to passive conditions (*p* < 0.05). There was also an angle (F_2,28_ = 16.26, *p* = 0.001, η^2^ = 0.538) effect as the SWE modulus (collapsed for all conditions and muscles) was greater at 0° compared to 45° and 90° (*p* < 0.05). To better illustrate the influence of passive condition on the modulus, we calculated the percentage increase in the modulus from 90° to 0°, which was 18.93 ± 8.93% for ES, 26.01 ± 11.3% for MF, 103.32 ± 37.3% for ST, and 137.01 ± 39.1% for SM.

**Figure 2:**
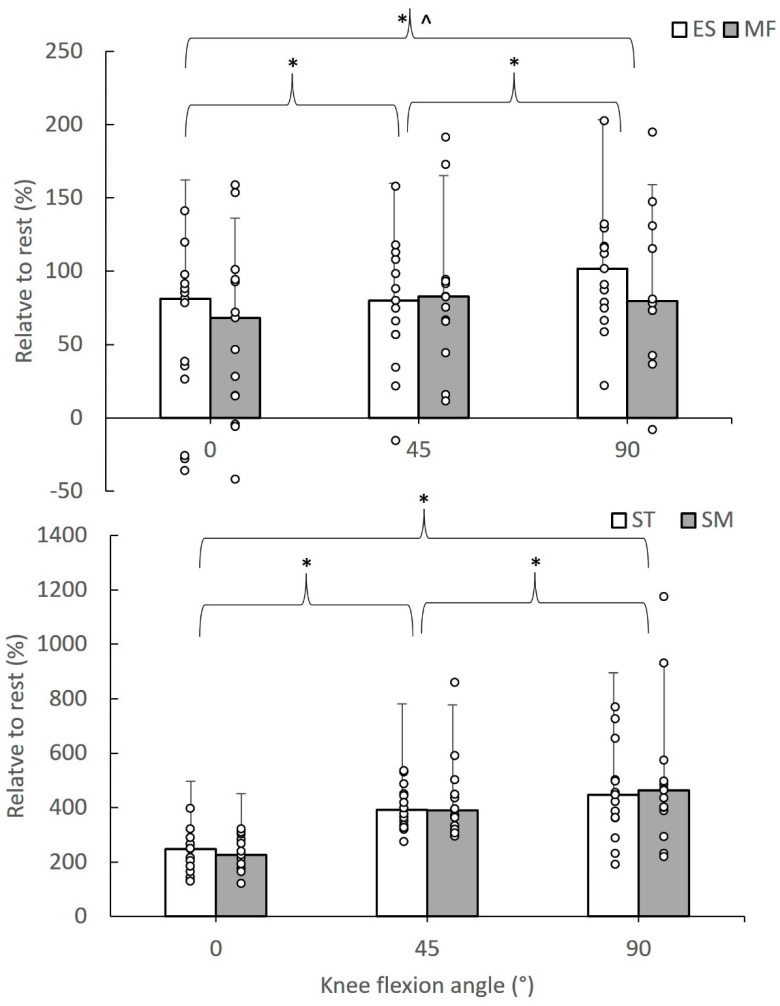
Median value of the relative SWE modulus of erector spinae (ES), multifidus (MF), semitendinosus (ST), and semimembranosus (ST) values during active contractions of the hamstrings at 0, 45, and 90° knee flexion angles. Error bars indicate the interquartile range, and circle dots are individual case values (* indicates a statistically significant difference between angles at *p* < 0.05; ^ indicates a statistically significant difference with ST and SM values, *p* < 0.05).

Figure 2 shows the median value of the relative SWE modulus during active hamstring contractions at different knee flexion angles, highlighting significant differences between angles and muscles. In particular, the median (interquartile range) of the relative SWE modulus during active tests ranged from 68.11 (86.29) to 101.69 (54.33)% for the paraspinal muscles and from 225.94 (114.72) to 463.16 (185.16)% for the hamstrings. Friedman tests showed that the relative SWE modulus differed between muscles at 0° (chi^2^ = 36.20, df = 3, *p* = 0.001), at 45° (chi^2^ = 36.00, df = 3, *p* = 0.001), and at 90° (chi^2^ = 37.00, df = 3, *p* = 0.001). Further comparisons between pairs of values showed that the relative SWE modulus of the ES and MF was lower than the corresponding SM and ST values (*p* < 0.05). For each muscle, the relative SWE modulus was greater at 90° than the remainder angles, while the modulus at 45° was greater than the 0° value (*p* < 0.05).

### 3.1. Correlation between Relative SWE Modulus of Paraspinal and Hamstring Muscles

There was a significant (*p* < 0.05) moderate to high correlation between the ES and MF relative SWE modulus at 0° (r = 0.943), 45° (r = 0.650), and 90° (r = 0.586). Moderate to high correlation coefficients were also found between ST and SM, with r values being 0.557, 0.588, and 0.675 at 0°, 45°, and 90°, respectively (*p* < 0.05). The ES relative modulus had a moderate to high significant correlation with ST (r = 0.807, 0.664, and 0.789, at 0°, 45°, and 90°, respectively), while the correlation between ES and SM relative modulus was significant at 0° (r = 0.661) and 45° (r = 0.764) but not at 90° (r = 0.446, *p* > 0.05). The MF relative modulus had moderate to high correlation with ST, which was significant at 0° (r = 0.661) and 90° (r = 0.679) but not at 45° (r = 0.425). The correlation between MF and SM relative modulus was moderate and statistically significant at 0° (r = 0.689) but not at 45° (r = 0.396) and 90° (r = 0.404, *p* > 0.05).

### 3.2. Muscle Activation

Table 2 presents the RMS for various testing conditions. There was a non-statistically significant three-way interaction effect on normalized RMS values (*p* > 0.05). The condition by muscle (F_2,18_ = 63.33, *p* = 0.0001, η^2^ = 0.976) interaction effect was statistically significant. Post-hoc Tukey tests showed that in the passive condition, there were no significant differences in RMS between muscles, whilst in the active condition, ST/SM activation was greater than ES and MF activation, and MF had a greater RMS value than ES (*p* < 0.05). All muscles had greater RMS values in the active compared to the active condition (*p* < 0.05). The angle by the muscle (F_4,36_ = 3.31, *p* = 0.021, η^2^ = 0.268) interaction effect was also statistically significant. Post-hoc Tukey tests showed that ST/SM RMS at 0° and 45° was greater than that recorded at 90°, while MF and ES RMS at 0° was greater than that recorded at 90°.

## 4. Discussion

In this study, we hypothesized that passive knee flexion would change not only the stiffness of the hamstrings but also the stiffness of the paraspinal muscles. The results partly confirmed this hypothesis, as passive knee flexion increased the SWE modulus of all muscles (Table 1). As expected, the relative increase in stiffness from 90° to 0° was much greater for the hamstrings (>100%) than the paraspinal muscles (18–27%). Based on our research in the literature, there are no previous reports on the effect of passive knee extension/flexion on paraspinal muscle stiffness. These findings provide further support to previous suggestions regarding which exercise influences not only the muscles that exert forces around the involved joint (s) but also whether it can affect other areas [7]. These non-localized effects have been attributed to the myofascial anchor points, which allow the transmission of in-series forces along these paths [6,9,39]. Previous studies have reported that the biceps femoris long head interfaces to the sacrotuberal ligament, which in turn has connections with the thoracolumbar fascia, which surrounds the paraspinal muscles [4,9,10,40]. In a cadaveric study, traction of the biceps femoris muscle was associated with movement of the thoracolumbar fascia [9]. While there is no previous research that has examined the influence of knee flexion angle on paraspinal muscle SWE modulus, some studies have provided significant associations between pelvic motion and gastrocnemius fascia [40] and between ankle dorsiflexion and SM fascia displacement [10]. An increase in stiffness due to a greater activation is highly unlikely, as we have found no differences in muscle EMGs between different joint angles (Table 2). Hence, our results support these findings and suggest that changes in knee flexion angle, which causes changes in hamstrings’ length [41], promote an increase in ES and MF stiffness.

When participants performed submaximal hamstring contractions, there was an increased stiffness in both paraspinal and hamstring muscles (Table 1), thus confirming our hypothesis. The increase in hamstring SWE modulus during contraction is in line with previous studies [23,24], and it can be attributed to the almost linear relationship between muscle SWE modulus and torque/force, which is exerted by the knee flexors [23]. There are various factors that might explain the increase in the SWE modulus of ES and MF during active knee flexor contractions. In the present study, we found a moderate to strong association between paraspinal and hamstring change in the SWE modulus from rest to active contraction. Hence, the present results add to previous experimental data, which support the suggestion that long chains of myofascial tissues create paths that allow force transfers between various body parts [6]. A second factor that may also be responsible for the results is greater neuromuscular activation of the paraspinal contracted musculature. Our results showed that activation of the ES and MF increased up to 20–24% during knee flexion contractions (Table 2). One may suggest that an elevated co-contraction of the lumbar spine muscles and the hamstrings may serve to facilitate lumbar, pelvic, and lower limb movement. Some evidence, for example, indicated that maximal knee flexion is partly related to an increased intrabdominal pressure [42], which, in turn, can increase paraspinal muscles’ activation [43]. Furthermore, it is known that during isometric fatiguing trunk extension contractions, there is co-activation of the lumbar spine muscles, the gluteus maximus, and the hamstrings, with the role of the hamstrings increasing as the test progresses [35]. In addition, an elevated activation of the gluteus maximus [44] and, hence, the transmission of forces to the lumbar muscles and fascia is possible [6,9,39].

No differences in the SWE modulus between the paraspinal muscles (ES and MF) were found (Table 1) even though MF exceeded ES activation (Table 2). The SWE modulus results are in line with some studies [27,29] but not with others [25,28]. Differences between studies can be attributed to variations in the subject positioning during the test as well as the probe location. In some studies [29] (including ours), ES and MF stiffness were measured 4 cm laterally from L4, while others placed the probe closer to the midline [25] or measured ES stiffness in a different position compared to MF [28]. The absence of differences in stiffness between ES and MF could be related to the type of tests examined in the present study. In particular, the test did not involve passive or active lumbar movement, and hence, no direct activation of the ES and MF was performed. The thoracolumbar fascia and the ES aponeuroses wrap and connect with the ES and MF muscles, forming a complicated mechanical system [9,45]. Consequently, if changes in hamstring stiffness are transmitted to the lumbar fascia, then a change in lumbar fascia stiffness could have caused a similar response (i.e., increase in stiffness) from the ES and MF.

No differences in the SWE modulus between ST and SM were also found, which is in line with a recent study [22] that examined changes in knee angle with the hip in a neutral position. Our results, however, are not in line with previous studies that reported greater ST stiffness than SM during knee flexion contractions [23,24]. The reason for this difference is unclear, but it may be related to differences in probe position, such as the protocol that was used to obtain the measurements between studies. Based on the evidence reported by Mendes et al. [24], differences in the SWE modulus between the hamstrings appear to be evident about 5–8 s after initiation of the contraction. Hence, it is highly possible that we did not detect such differences, as in our study, the participants performed 5 s contractions.

There are some limitations of this study. First, in the present study, we examined physically active young males with no history of musculoskeletal pain or recent injuries. The results may differ if people with lumbar pain or hamstring injury or different ages are examined. Second, like every study, the use of a greater sample size would have yielded more robust results. Nevertheless, the magnitude of the effect size (η^2^ > 0.327) was very high, which suggests that the risk of making a type 2 statistical error (false-negative) is highly unlikely. Third, the SWE modulus values are specific to the location of the probe along the muscle during the test [21,37]. Further, even though the US probe was oriented so that it was parallel to fascicle orientation, changes in fascicle position relative to the skin during tests may have resulted in an angulation between the probe and the fibers, thus altering elastography measurements. Further, it is possible that activation of other muscles, such as the gluteus maximus and the biceps femoris long head [9], and pelvic movement during contraction [46], might have increased paraspinal activation. Even though we stabilized the pelvis and the hip during the test, the influence of various muscles that surround these areas guarantees further research. In addition, EMG activation and SWE measurements were not obtained from exactly the same positions, and this might have impacted direct relationships between the two datasets. It should be mentioned, however, that with the current technology, this is a difficult task. Finally, another limitation is the interindividual variability in response to exercises, which could influence the generalizability of the results.

The present findings have some implications. It seems that passive changes in knee flexion angle from the prone position cause about a 25% increase in the paraspinal muscles’ stiffness. This is in line with previous findings or suggestions that interventions such as myofascial release applied on various areas along the superficial back line (which extends from the thoracolumbar to the plantar area) increased hamstrings’ flexibility [5]. Similarly, performing submaximal knee flexion efforts (leg curls, for example) increases paraspinal muscle stiffness. This provides support to previous results from mathematical simulations, which show that contraction of the ES is associated with stretching of the hamstring muscles [11], as well as experimental findings which show a significant association between back extensor length and hip extensor strength in individuals with low back pain [16]. These findings suggest that paraspinal muscle stiffness can be modulated through specific hamstring exercises, which is relevant for designing rehabilitation programs for patients with low back pain and vice versa.

### Conclusions

The SWE modulus of the paraspinal and hamstring muscles increased about 25% and 100–125%, respectively, when the knee joint changed position from 90° to 0° of knee flexion. During submaximal hamstring contractions, the SWE modulus of all muscles increased significantly, and there was a moderate or strong association between the paraspinal and hamstring muscles. These results support previous suggestions that transmission of forces through myofascial connections, in addition to neural control synergies, between the lumbar spine and the lower limbs takes place, which allows to exercise muscles in one area of the body and affects the stiffness of the muscles in another area. These results support the integration of specific hamstring and lumbar exercises in training and rehabilitation programs to improve muscle stiffness and prevent injuries or reduce pain incidents.

## Figures and Tables

**Table 1:** Mean (±standard deviation) shear wave elastic modulus (kPa) of the erector spinae (ES), multifidus (MF), semimembranosus (SM), and semitendinosus (ST) during active and passive tests from 0°, 45°, and 90° angles of knee flexion.

Knee Angle	ES	MF	ST	SM	Between-Muscle and Condition Differences, *p* < 0.05
	Passive				
0	12.44 ± 4.45	13.35 ± 6.12	22.01 ± 4.68	21.57 ± 5.22	ES, MF < ST, SM
45	10.86 ± 5.41	10.22 ± 4.61	12.82 ± 3.45	12.25 ± 2.80	
90	9.89 ± 5.83	11.33 ± 2.68	10.85 ± 4.23	9.16 ± 2.93	
	Between-angle differences, *p* < 0.05				
	0° > 45°, 90°				
	Active				
0	18.99 ± 6.11	20.65 ± 11.31	71.21 ± 13.88	70.20 ± 14.29	ES, MF< ST, SM Active > passive
45	18.12 ± 7.15	20.27 ± 11.17	63.80 ± 17.46	63.55 ± 17.77	
90	19.21 ± 10.11	21.19 ± 6.80	58.15 ± 17.39	50.91 ± 15.04	
	Between-angle differences, *p* < 0.05				

**Table 2:** Mean (±SD) normalized EMG (percentage of maximum voluntary contraction) of the erector spinae (ES), multifidus (MF), and semimembranosus/semitendinosus (SM/ST) during passive and active tests.

Knee Angle (°)	ES	MF	ST/SM	Between-Muscle and Condition Differences, *p* < 0.05
	Passive condition			
0	2.79 ± 1.19	7.11 ± 1.90	4.09 ± 1.62	
45	2.80 ± 1.71	6.67 ± 1.94	9.68 ± 5.18	
90	3.85 ± 2.89	6.44 ± 3.39	5.48 ± 2.78	
	Active condition			
0	19.54 ± 6.69	24.23 ± 7.33	71.91 ± 20.48	ST/SM > ES, MF MF > ES Active > Passive
45	11.82 ± 6.63	18.07 ± 9.17	69.02 ± 22.31	
90	9.81 ± 4.50	14.40 ± 3.83	52.75 ± 16.71	
Between-angle differences, *p* < 0.05	0 ° > 90°	0 ° > 90°	0° > 90° 45° > 90°	

## Data Availability

Data are available are available at https://data.mendeley.com/datasets/jpdwh3ny8z/1 (accessed on 15 July 2024).
